# Prevalence and associated factors of noise-induced hearing loss among workers in Bishoftu Central Air Base of Ethiopia

**DOI:** 10.1038/s41598-024-56977-4

**Published:** 2024-05-10

**Authors:** Ashenafi Hailu, Birhanu Zeleke, Zeberihe Ermias, Fasil Kenea Duguma, Sara Dula, Samson Wakuma Abaya, Seblework Mekonen Shegen, Gudina Terefe Tucho, Tariku Neme Afata

**Affiliations:** 1 Department of Environmental Health Science and Technology, Jimma University, PO box 373, Jimma, Ethiopia; 2 Defense University College of Health Sciences, Bishoftu, Oromia region Ethiopia; 3 Dambi Dollo Teachers College, Dambi Dollo, Oromia region Ethiopia; 4 School of Public Health, Addis Ababa University, Addis Ababa, Ethiopia; 5 Department of Water and Public Health, Ethiopian Institute of Water Resources, Addis Ababa University, Addis Ababa, Ethiopia

**Keywords:** Noise, Noise-induced hearing loss, Hearing conservation program, Noise level, Natural hazards, Health occupations, Risk factors, Signs and symptoms

## Abstract

Excessive occupational exposure to noise results in a well-recognized occupational hearing loss which is prevalent in many workplaces and now it is taken as a global problem. Therefore, this study aims to assess the prevalence of noise-induced hearing loss and associated factors among workers in the Bishoftu Central Air Base in Ethiopia. An institutional-based cross-sectional study was conducted among 260 central air base workers through face-to-face interviews, an environment noise survey, and an audiometric test for data collection. Data were entered by Epi-data version 3.1 and SPSS was used to analyze the data. Finally, a statistical analysis such as descriptive and binary logistic regression analysis was applied. A P-value < 0.05 at 95% CI was considered statistically significant. The overall prevalence of noise-induced hearing loss and hearing impairments was 24.6 and 30.9%, respectively. The highest prevalence of noise-induced hearing loss was recorded for workers who were exposed to noise levels greater than 90 dBA. Out of 132 workers exposed to the average noise level of 75 dB A, only 5% of workers were affected with noise-induced hearing loss, while 128 workers exposed to an average noise level equal to or greater than 90 dB A, 19.6% of workers were identified with noise-induced hearing loss. Regarding sex, around 21.9% of male workers were identified with noise-induced hearing loss. Workers who were exposed to a high noise level workplace previously or before the Central Air Base workplace were five times (AOR = 5.0, 95% CI 1.74–14.36) more likely affected by noise-induced hearing loss than those workers not previously exposed. Those workers who were exposed to greater or equal to 90dBA noise level were 4.98 times (AOR = 4.98, 95% CI 2.59–9.58) more likely to be exposed to noise-induced levels than those who were exposed to less than 90dBA noise level. Moreover, male air base workers were 3.5 times more likely exposed to hearing impairment than female workers (AOR = 3.5, 95% CI 1.01–12.0). This study identified that the prevalence of noise-induced hearing loss and hearing impairments was significantly high. So implementation of a hearing conservation program, giving noise education, and supplying adequate hearing protective devices (HPDs) are essentials.

## Introduction

Noise is an excessive or unwanted sound that potentially results in annoyance and/or hearing loss^1^. Noise-induced hearing loss (NIHL) is the result of multiple factors causing harm to the auditory systems after being exposed to loud noises in the workplace, outdoors, or during leisure activities^2–4^. Research has demonstrated that even low noise levels of 74 dB can result in temporary hearing loss and regular exposure to noise levels above 85 dB will result in irreversible hearing loss, primarily at high frequencies, as a result of damage to sensory hair cells in the cochlea's basal turn^5^. However, long-term hearing loss is contingent upon other factors such as noise intensity, time of exposure, individual susceptibility, and personal hearing protection use^6^.

Currently, the aviation industry noise has been acknowledged as a problem in terms of the environment and operations^2^. The constant noise produced by aircraft engines is a byproduct of civilization that aircrew members encounter daily^7,8^. For the past 10 years, the evidence showed that aircraft noise exposure leads to increased risk for poorer cardiovascular health has increased by 7 to 17% for a 10 dB increase in aircraft noise exposure^9^.

Especially, military pilots are subjected to a potentially hazardous level of noise compared to commercial jet pilots, which could be a significant risk factor for irreversible hearing damage^10,11^. However, different aircraft types have different noise levels and frequencies. The noise from the aviation industry originates from three main sources: aerodynamic noise, engine/mechanical noise, and noise from aircraft systems. Noise can also be produced by aircraft equipment, transmission systems, propellers, rotors, hydraulic and electric actuators, air conditioning and cabin pressurization systems, alert systems, and communications equipment^12,13^.

Additionally, noise-induced hearing loss (NIHL) is a major problem among personnel serving in the armed forces as their profession exposes them routinely to extreme noise levels that affect their hearing. The soldiers, airmen, and sailors are exposed to high levels of noise produced by aircraft, ships, heavy mechanical transports, and the weaponry they use^2,14^.

Moreover, several studies have reported that the predictive factors related to NIHL were being male sex, higher age, duration of exposure or service year, noise intensity level, exposure to an explosion, vibration, smoking, ceruminous occlusion of the external auditory canal, and utilization of hearing protective devices as preventive factors^14–16^.

Furthermore, a few countries have published studies on the prevalence of noise-induced hearing loss in military aviation industries. The studies conducted in the Indian Air Forces audiometric notch revealed that an overall prevalence of NIHL was 22.9%^2,15^, while, in the maintenance crew of the Institute of Aviation Medicine of the Royal Malaysian Air Force, it was 41.2%^17^. A similar study conducted by the Israel Air Force showed that the prevalence of NIHL was 57.5% among those aged 50 years^18^. Moreover, a study conducted in Malaysia, Saudi Arabia King Kahilid, and Jomo Kenyatta international airport workers NIHL were 33.5%^19^, 33.5%^20^, 16%^21^, respectively.

However, in Ethiopia, there is no study conducted in aviation industries. Still, there was a study conducted in the textile and metal industry with the prevalence rate for the NIHL was 34 and 22%, respectively^16,22^. Although aviation workers in Ethiopia are exposed to noise every day the level of noise exposure and its health effects are not known so understanding the noise level, prevalence, and factors related to noise-induced hearing loss is essential for effective noise prevention and control strategy. Therefore, this study aims to assess the level of noise-induced hearing loss and associated factors among workers in the Bishoftu Central Air Base of Ethiopia.

## Results

### Socio-demographic characteristics of respondents

Out of 264 samples needed, 260 were included for the assessment of NIHL and 262 for the general assessment of hearing impairment study. Two of the workers with tympanic rupture or perforated eardrums differentiated with otoscopic physical examination were excluded from the study of NIHL but included in the assessment of general hearing impairment. The response rate was 98.5% for NIHL and 99.2% for hearing impairment. The mean age of workers was 30 ± 7 years. The majority of age categories were between 18 and 27 (47.7%) years with service of 1–10 (73.8%) years. Most of the participants of the workers were males (82.0%) and single 161 (61.9%). Most of the educational level study participants were Technical/ college diploma 147 (56.5%) followed by degree and above 80 (30.8%). Of those participants, 254 (97.7%) were military personnel with the job category of maintenance technicians115 (44.2%), flight and ground technicians 73(28.1%) (Table 1).

**Table 1:** Socio-demographic characteristics of respondents of Bishoftu Central Air base workers of Ethiopia.

Characteristics		n	(%)
Sex	Male	226	86.9
	Female	34	13.1
Age in years	18–27	124	47.7
	28–37	91	35
	38–47	36	13.8
	> 47	9	3.5
Service in years	1–10	192	73.8
	11–20	50	19.2
	> 20	18	7
Religion	Orthodox	184	70.8
	Muslim	14	5.4
	Protestant	58	22.2
	Catholic	3	1.2
	Other	1	0.4
Marital status	Single	161	61.9
	Married	99	38.1
Educational status	Write and read	1	0.4
	Primary school 1–8	7	2.7
	Secondary 9–12	25	9.6
	Technical/diploma	147	56.5
	Degree and above	80	30.8
Category of the participants	Civil	6	2.3
	Military	254	97.7
Working department	Security	23	8.9
	Cisinia	12	4.6
	Su-27 jet	35	13.5
	Transport helicopter	29	11.2
	Transport airplane	18	6.9
	L-39 jet	19	7.3
	Logistics	10	3.8
	Garage	25	9.6
	Daily maintenance (DM)	89	34.2
Job category	Pilot	8	3.1
	Mechanic technicians	116	44.6
	Electronics and communication maintenance	17	6.5
	Flight and ground technician	73	28.2
	Navigation crew	8	3.1
	Armament	6	2.3
	Driver	5	1.9
	Welder	4	1.5
	Security	23	8.9

*n* number.

### Personal and health-related history of the workers

The study revealed that 19 (6.2%) workers were previously exposed to noisy work area before they join to Bishoftu central air base. Additionally, 94.6% of the workers were participated in military services, and out of them 18% of the workers were exposed to noises of gunshots and different types of explosions in battle wars. Regarding to the complaining of their hearing capacity, 15% and 14.2% of the workers fill as they developed tinnitus and hearing problems, respectively (Table 2).

**Table 2:** Personal and health-related history of Bishoftu Central Air Base force workers of Ethiopia.

Characteristics of the variables		n	%
Previous work in another noisy area	Yes	19	7.3
	No	241	92.7
Ever been grinding or welding metal	Yes	37	14.2
	No	223	85.8
Ever been in military service	Yes	246	94.6
	No	14	5.4
If the military is ever exposed to a gunshot or explosion in battle war	Yes	46	18
	No	200	82
Ever been trained/given service as a pilot	Yes	10	3.8
	No	250	96.2
If yes type of plane	L – 39	1	10
	Transport helicopter	6	60
	Su – 27	3	30
Had a pre-employment hearing test	Yes	109	41.9
	No	151	58.1
history of hearing loss before this job	Yes	6	2.3
	No	254	97.7
Ever had ear discharge trauma or infection	Yes	22	8.5
	No	238	91.5
Ever had a head injury	Yes	14	5.4
	No	246	94.6
Had a broken ear drum identified by a health professional (self-report)	Yes	5	1.9
	No	255	98.1
Had a cold in the last fortnight (last 2 weeks)	Yes	37	14.2
	No	223	85.8
Have an ear problem now	Yes	42	16.2
	No	218	83.8
Hearing problem now	Yes	37	14.2
	No	223	85.8
Otitis	Yes	8	3.1
	No	252	96.9
Filling of hearing difference b/n two ears	Yes	45	17.3
	No	215	82.7
Filling of tinnitus in the ear or head	Yes	39	15
	No	221	85
A family lost hearing before the age of 50 years	Yes	6	2.3
	No	254	97.7
Hypertension identified by a health professional	Yes	10	3.8
	No	250	96.2
Diabetic identified by a clinician	Yes	2	0.8
	No	258	99.2
Vibration in your section	Yes	139	53.5
	No	121	46.5
Body mass index	Underweight (below 18.5)	13	5
	Normal weight (18.5–24.9)	186	71.8
	Overweight (25–29.9)	59	22.8
	Class I obesity (30–34.9)	1	0.4

*n* number.

### Use of personal protective devices, hearing loss perception, and training

Regarding the use of hearing protective devices, 89.6% of the workers believed as hearing protective devices (HPDs) are beneficial, 6.9% didn`t know its purpose and 3.5% of them assumed as it was not beneficial. Even 17.3% of the workers thought as HPDs did interfere with their hearing ability or impose danger on their hearing capacity. From the participants, only 86 (33.2%) of the participants were used personal hearing protective devices, of those 62.79% used ear plugs, and 37.21% ear muffs. In terms of frequency of using hearing protective devices, only 15.9% of them used always, and 84.9% of them used sometimes. Workers perceptions in terms of noise showed that 83.5% of them perceived as noise can induce hearing loss and 80.8% considered their current work place was noisy and harm their hearing ability but 8.1% of them didn`t know whether harm or not, 11.2% of the workers explained that as their current work place didn`t impose any harm on their hearing capacity. In the case of safety training or education, 49.8% of the participants didn’t get safety training with different durations. Regarding noise`s health effect, 96.2% of the workers didn`t get training on the effect of noise on their health at their workplace. Furthermore, about the law or rule that obligates the workers they use personal protective equipment, only 17.3% said as a rule was available at their institution but 3.5% of the participants didn`t know and 79.2% of them believe that rules and regulations that enforce the workers didn`t found in their institution.

### Environmental noise measurements

The noise level of different planes and departments at personal exposure was measured at the maximum idle running of motors and maximum running of different jets, and plane motors at the place of pilot exposure, and workers exposure. The study revealed that the sound level of transport airplane L-100 emitted at idle was 123 dBA and at maximum power emitted was 128 dBA at the ground technician's site of exposure but at the site of pilot exposure was 76 dBA at ground running of four engines.

The other transport helicopter M-17 sound level measurement revealed a maximum level of 109 dBA externally but 102 dBA passenger`s seat and at idle engine run the maxim sound level was 99 dBA, at the position of a pilot the maximum sound level captured was 96 dBA. Moreover, the Su-27 jet with two engines emitted 110 dBA at idle state and 128 dBA at maximum engine run-up but it was difficult to measure the internal part at the pilot site due to high production of high pressure and sucks the surrounding air once it started to run up the engine. Additionally, it was forbidden to reach the jet, and the other detail sound level of the plane was measured (Table 3).

**Table 3:** The maximum sound level measured by dBA at idle and maximum engine run-up of the planes and jets of Bishoftu Central Air Force of Ethiopia.

Type of plane	Max. sound level at idle engine run-up	Max sound level at max engine run-up	Pilot’s exposure at max engine
Transport airplane	123	128	76
L-39 jet	105	115	98
Cesena	81	110	90
Transport helicopter	99	109	96
Su-27 jet	110	128	–

*NB* The measurement was taken at the position of the exposure of the workers.

### Prevalence of noise-induced hearing loss (NIHL) and associated factors

This institutional-based study attempted to assess the prevalence, the magnitude of noise emitted, and determinant factors of noise-induced hearing loss among Central Air Base workers. The overall prevalence of noise-induced hearing loss was 24.6%. This prevalence comes from bilateral (16.9) and unilateral (7.7%) noise-induced hearing loss. The highest prevalence noise level was recorded for workers who were exposed to noise levels greater than 90 dBA. Out of 132 workers exposed to the average noise level of 75 dB A, only 5% of workers were affected with noise-induced hearing loss. Whereas among 128 workers exposed to an average noise level equal to or greater than 90 dB A, 19.6% of workers were identified with noise-induced hearing loss. Regarding the sex, around 21.9% of male workers were affected with noise-induced hearing loss. Factors associated with the prevalence of noise-induced hearing loss were working in a noisy area before an air force (in the metal industry, textile industry, creasier and wood factory), workers` feeling a hearing difference between their right and left ears, workers` perceive themselves as they have hearing problems, and workers exposed to noise level above or equal 90 dB A have a p-value less than 0.05 in bivariate analysis and were a candidate for multivariate logistic regression but the level of working in noise area before the air force, and noise level (dBA) were significant associations with noise-induced hearing loss in multivariate logistic regression analysis (Table 4).

**Table 4:** Prevalence of NIHL and associated factors among Bishoftu Central Air Base workers of Ethiopia.

Characteristics of the variables		NIHL		COR (95% CI)	AOR (95% CI)
		Yes (%)	No (%)		
Worked in noise before an air force	Yes	9 (3.5)	10 (3.8)	3.04 (1.18–7.87)**	4.7 (1.61–13.64)***
	No	55 (21.2)	186 (71.5)	1	1
Have hearing differences b/n the two ears?	Yes	18 (6.9)	27 (10.4)	2.45 (1.24–4.83)**	0.4 (0.16–1.02)
	No	46 (17.7)	169 (65)	1	1
Feel hearing problem	Yes	14 (5.4)	23 (8.8)	2.1 (1.01–4.39)**	0.95 (0.35- 2.62)
	No	50 (19.2)	173 (66.5)	1	1
Noise level (dBA)	≥ 90	43 (16.9)	65 (25)	4 (2.26–7.52)**	5.2 (2.68–10.18)***
	< 90	21 (8.1)	131 (50.4)	1	1

COR crude odd ratio, *AOR* adjusted odd ratio, *CI* confidence intervals.

**Significant at P < 0.05 bivariate analysis, ***Significant at P < 0.05 multivariate analysis.

### Prevalence and associated factors of hearing impairment (HI)

The overall prevalence of hearing impairment among the workers was 81 (30.9%) with 45 (17.2%) bilateral and 36 (13.7%) unilateral hearing impairments. Among the bilateral hearing impairment, slight impairment 23 (63.9%), moderate impairment 10 (27.8%), severe impairment 1 (2.8%), profound impairment 2 (5.6%), and from those unilateral right ear, slight impairment 36(78.3%) moderately impairment 8(17.4%) and profound hearing loss 2 (4.3%), left ear, slight impairment 35 (57.4%) moderate impairment 20 (32.8%), profound impairment 6 (9.8%).

Factors like level of sex, hearing the difference between two ears, hearing problems, tinnitus/ringing in the ear, and normal otologic examination have a *p*-value of less than 0.05 in bivariate analysis and were a candidate for multivariate logistic regression. However, due to potential con-founders, some variables were significant associations in the bivariate analysis but their significance disappeared in the multivariate analysis. Thus, sex and normal otologic examination were significant associations with hearing impairment in multivariate logistic regression analysis. The result revealed that male air base workers were 3.5 times more likely exposed to hearing impairment than female workers (AOR = 3.5, 95% CI 1.01–12.0). Moreover, those participants who have abnormal otologic examinations (having waxy, otitis, or tympanic rupture) were 1.9 times more likely exposed to hearing impairment than those who have normal otologic examinations (AOR = 1.9, 95% CI 1.02–3.54) (Table 5).

**Table 5:** Prevalence and associated factors of hearing impairment (HI) among Bishoftu Central Air Force workers, Ethiopia.

Characteristics		HI		COR (95% CI)	AOR (95% CI)
		Yes (%)	No (%)		
Sex	Male	78 (29.8)	150 (57.2)	5.4(1.59–18.13)**	3.5 (1.01–12.0)***
	Female	3 (1.1)	31 (11.8)	1	1
Hearing difference b/n two ears?	Yes	25 (9.5)	22 (8.4)	3.2 (1.69–6.17)**	1.6 (0.7–3.93)
	No	56 (21.4)	159 (60.7)	1	1
Feel hearing problem	Yes	20 (7.6)	17 (6.5)	3.2 (1.56–6.44)**	2 (0.83–4.9)
	No	61 (23.3)	164 (62.6)	1	1
Having tinnitus/ringing in the ear	Yes	23 (8.8)	18 (6.9)	3.6 (1.81–7.13)**	2.2 (0.93–5.25)
	No	58 (22.1)	163 (62.2)	1	1
Normal otologic examination	No	28 (10.7)	36 (13.7)	2 (1.19–3.82)**	1.9 (1.02–3.54)***
	Yes	53 (20.2)	145 (55.3)	1	1

*COR* crude odd ratio, *AOR* adjusted odd ratio, *CI* confidence intervals.

**Significant at P < 0.05 bivariate analysis, ***Significant at P < 0.05 multivariate analysis.

## Discussion

This study revealed that noise-induced hearing loss (NIHL) among central air base workers was high. The overall prevalence of noise-induced hearing loss was 24.6%. The current noise-induced hearing loss was higher than the studies done at Saud Arabia King Khalid International Airport (12%)^20^, and Jomo Kenyatta International Airport (15.3%)^21^. Similarly, the prevalence of noise-induced hearing loss was higher than the study conducted in India (22.9%)^14^, and lower than the study conducted in Malaysian 41.2%^17^, and Israel Air Force^18^. These differences may be due to the different levels of noise emitted, different hearing conservation programs, different exposure levels of the workers, and the awareness level of the countries on the impact of noise. Additionally, the lack of supply of personal protective devices and enforcement of rules and regulations of safety in this country is not as applicable.

According to this study, the overall prevalence of hearing impairment among the workers was 30.9% (95% CI 26.0–36.6) with 17.2% bilateral and 13.7% unilateral hearing impairments. which was lower than the study conducted by the India Air Force^23^ but higher than the study conducted among workers at Malaysia Airport^24^ and Saud Arabia King Khalid International Airport workers^25^. This is due to noise-induced hearing factors like treating otitis infections and lack of regular follow-up.

Additionally, the study revealed that 41.5% of workers were exposed above 90 dBA with no specified period because of the nature of the work. Even some planes and jets like the Transport airplane (Antonovo), Su-27, Transport helicopter, and daily maintenance Garage measured at the workers` positions of different places revealed that high exposure to noise level due to the exposure of workers was not bound with the time of exposure standards rather it depended on the nature of work. This fails the Ethiopia occupational safety and health directives regulation on the level of noise and respective time exposure^26^. Another study conducted by Malaysian and Indian airport workers revealed that the highest noise level was recorded at aircraft starting its engine at different distances as well as during day and night time^15,26^. The main difference in noise levels might be due to the difference in planes, jets, and machines' sound emissions levels.

Moreover, the other factor that was significantly associated with NIHL in this finding was previous exposure of the workers to noisy work environments like metal and woodwork industries. Once the hair cells become damaged it becomes permanent and individuals exposed previously became positive for NIHL^27^.

Additionally, this study revealed that low utilization of hearing protective devices (HPDs) among air-base workers was exposed to NIHL. Due to a lack of safety training, negligence, and lack of awareness of the use of PPEs. The study conducted in Saud Arabia King Kahild and India International Airport revealed that those workers who never used HPDs regularly developed NIHL^14,20,22,28^.

Moreover, The result revealed that male air base workers were 3.5 times more likely exposed to hearing impairment than female workers (AOR = 3.5, 95% CI 1.01–12.0). This result aligned with Studies conducted in Kenya and the USA^21,29,30^. The reason may be due to the high exposure of males to noise than females especially in developing countries due to the nature of the work environments.

Finally, those participants who have abnormal otologic examinations (having waxy, otitis, or tympanic rupture) were 1.9 times more likely exposed to hearing impairment than those who have normal otologic examinations (AOR = 1.9, 95% CI 1.02–3.54). A similar study revealed that participants who had abnormal otologic examinations were more exposed to hearing impairment than the other workers^31–33^. This risk factor may occur due to the occlusion or blockage of the ear canal.

The study has several drawbacks. Lack of generalizability with the Ethiopian Air Force since it was done only on the central base of the Air Force. Power interruption during audiometric measurements, weight balance, and height measurements since the materials were digital and needed electric power. Furthermore, running the engine at the maximum was so difficult because of the nature of the plane and its fuel cost was so expensive, and senior staff was reluctant to participate in the study.

## Conclusion

Workers of the Central Air base especially the flight line were exposed to high levels of occupational noise that could induce immediate hearing loss. Specifically, the magnitude of noise emitted by jets like Su-27, L-39, transport helicopters, transport airplanes (Antonovo), and Cesena was high when compared with MOLSA and OSHA standards of noise level exposure against permissible time exposure even if the workers were not continuously exposed to the noise. The prevalence of hearing loss was significantly high, especially for those workers exposed to noise levels above 90 dBA. The supply of HPDs and safety training related to noise was low. So Implementation of a hearing conservation program, giving noise education, and supplying adequate HPDs are essentials.

## Materials and methods

### Study setting

The institution-based cross-sectional study design was conducted at the Ethiopian Central Air Force Base, which is found in Bishoftu town, Ada’a district, Oromia regional state between June to July 2018. The study area was 50 km away from the capital city, Addis Ababa and it was established in 1966 E.C.

### Study design

A cross-sectional study was conducted on 260 participants of Central Air Force base workers from June to July 2018 in central Ethiopia. During the data collection, the purpose of the study was explained to each participant. Written informed consent was obtained from each participant and participation in the study was completely voluntarily. Confidentiality was granted for the information collected from each study participants.

### Sample size determination

The sample size was determined using a single population proportion formula considering the p prevalence of NIHL 22% among Akaki basic metal industry workers^16^. A 95% confidence interval and a 5% margin of error were used to calculate the sample size. The sample size was determined using a single population proportion formula: $\frac{{(Z\alpha /2)}^{2} (p)(q)}{{(SE)}^{2}}$= $\frac{{(1.96)}^{2}( 0.22)(0.78)}{{(0.05)}^{2}}$  = 264, by considering a 10% non-response rate, the total sample size was found to be 290.

where n = Sample size, P = prevalence of NIHL in Ethiopia, Akaki basic metal industry 22%, d^2^ = marginal error = 5%, Zα/2: standard for Z score is 1.96 that corresponds to a 95% confidence level.

The total number of workers found in the central air base was estimated to be around three thousand. Using the reduction formula; N = n/(1 + n/N), the final sample size was calculated to be 264. During sample determination the Akaki Basic Metal Industry was taken as reference because of the reasons that workers in the air base and metal industry are almost similar in their socio-economic status income, behavior, biological, age, and military backgrounds are similar. Additionally, we considered also noise physical hazard causes workers exposed to high noise levels whether in the metal industry or aircraft it causes hearing losses even if there are differences in the magnitude of hearing losses among the groups.

### Operational definitions

#### Noise-induced hearing loss

Noise-induced hearing loss is sensory deafness caused by long-term exposure of the auditory system to a noisy environment (Threshold notch at 4 kHz or 6 kHz at least 25 dB HL (decibels hearing loss))^34^.

#### Hearing impairment

Hearing impairment is defined as a diminished or defective sense of hearing (when the average of 500 Hz, 1000 Hz, 2000 Hz, and 4000 Hz exceeds 25 dB HL (decibels hearing loss))^35^.

#### Worked in noise before air force

Those workers did previously in the metal industry, textile industry creasier, wood factory, etc^34^.

### Inclusion and exclusion criteria

The workers who served at least one year and above in the Air Force and had exposure to aircraft noise were included in the study. Finally, participants with a history of ruptured tympanic membranes were excluded according to the exclusion criteria.

### Sampling techniques

A total of 264 workers were needed. Then the workers were allocated proportionally to the size of the nine departments. The workers who served at least one year and above in the Air Force and those who had exposure to aircraft noise were selected by a simple random sampling method, using the workers’ rosters as a sampling frame from each department.

### Data collection

Basic socio-demographic and work-related data were collected using a pretested structured questionnaire which was performed in the metal fabrication industry found in Addis Ababa, Ethiopia. Otologic examination and other physical examinations (body mass index, blood pressure, and other vital signs) were performed by the clinician. The hearing level was determined by pure tone audiometric measurement using a digital recording audiometer known as Digital Diagnostic Audiometer AC50-D in the audiometer room of the surgeon clinic. The surgeon clinic audiometric test room is designed for audiometric testing with recommended background sound pressure levels for the air base institution. Workers have planned audiometric tests so that they can have a ‘quiet time’ of ideally 16 h before the audiometric test. The selected workers came to the surgeon's clinic and seat in the test room where he/she could not view audiometer controls while being tested. The audiometer was supplied with both standard audiometric headphones by putting the red color phone on the right ear and the blue on the left ear. By making sure that the diaphragm (center) of the earphone was directly over the ear canal, they were instructed to raise their hand when the tone was heard and to put down when the tone was no longer heard.

The testing frequencies were at 250, 500, 1000, 2000, 3000, 4000, and 8000 Hz. Then it started with the first familiarization tone at 1000 Hz and ended with the final presentation at 250 Hz. The tone was counted starting with the 30-dB HL familiarization. The testing order was followed with the standard method from 1000, 2000, 3000, 4000, and 8000 Hz, then repeating 1000 Hz to assess intra-test reliability and account for practice effects before proceeding to 500 and 250 Hz^36^. Generally, the bracketing method listed in ISO, 8253-1 was used to assess the hearing level. To identify the effect of noise, early or moderately advanced NIHL usually results in the typical ‘boilermakers’ notch at 4 kHz, with spread to the neighboring frequencies of 3 kHz and 6 kHz^37^, and some hearing recovery at 8 kHz was used^38^. The fact that frequencies around 4 kHz are most affected by noise, is due to the resonance frequency of the outer ear/ear canal as well as the mechanical properties of the middle ear^39^. But the cut-off point for reference we used was 4000 and 6000 Hz^15,38^. For hearing impairment, “at least 25 dB (A) in the better hearing ear (average over the frequencies 0.5, 1, 2 and 4 kHz)”. In grading of hearing impairment; those who hear less or equal to 25 dB A considered normal, 26–40 dB A slight impairment, 41–60 dB A moderate impairment, 61–80 dB A severe impairment, and above 80 dB A as profound impairment^38^.

The noise level was measured in workstations corresponding to each sampled worker's position by locating the microphone in the worker’s hearing zone (2 feet wide sphere) using a digital sound level meter model 840,029 Taiwan^40^. Measurements were recorded by holding the instrument at the upright position at a height of 1.5 m from the ground in the working environments of the workers to properly determine the noise level to which the workers are exposed using a digital sound level meter, adjusting the frequency weighting networks on (A) which are conformity to standards. The equipment meets OSHA requirements which meet IEC 61,672 class 2 and ANSIS 1.4 type 2 frequency and time weighting specifications, DIN 45,633, and JIS 1502 whose frequency weighting selectors A and C which were conformity to standards. The maximum level of noise was measured two times and the maximum average noise level was taken in one minute even additional 30 s were considered rather than mentioned in the noise manual which states as best practice to take two to three 30-s samples using the SLM and record the measured noise levels on the record sheet^41^. It has also time weighting (SLOW and FAST) which are dynamic characteristic modes. In this study, the FAST was selected to capture the peak noise level which may be occurred intermittently. The sound level meter was calibrated at 94 dBA before and after each measurement was conducted. Since the characteristic A weighting is simulated at the Human ear response, it is recommended to be used for environmental noise level measurements^42^. During measurement maximum effort was made to keep the microphone dry, avoid vibrations during measurement, and carry out measurements in steady temperature and humidity as much as possible as recommended.

### Data quality

The audiometer was calibrated at the outset of the study and recalibrated regularly using biological standards. Biological standards are healthy individuals on which the instrument is calibrated under the same environmental conditions or obtained by testing the hearing sensitivity of young, healthy adults and averaging the sound level at specific frequencies at which the tones were barely perceptible^22^. All audiometric tests were carried out in a quiet room within the surgeon clinic designed for audiometric tests before the interviewees entered their work shift to avoid the effects of temporary threshold shifts.

### Data analysis

Data was entered into Epi-Data version 3.1 and analyzed using the SPSS statistical package, version 21. Descriptive statistics were computed to display mean frequency and percentage. Bivariate and Multivariate logistic regression analyses were performed to identify factors associated with NIHL. The associations were described using an odds ratio with a 95% confidence interval. The logistic regression model was run to examine whether there was an association between the potential explanatory variables and outcome variables. The cutoff for statistical significance was p-value ≤ 0. 05. Moreover, crude and adjusted odds ratio with their corresponding 95% CI was used to measure the strength of associations between the independent and the outcome variables. For the Environmental measurement, the logarithmic results were analyzed and compared with the standards^42^.

### Ethical approval

An ethical approval letter was obtained from the Defence University College of Health Science Ethics Committee Review Board (IRB) on APR-20/2018 (MTC/set-20/T/08/30). The authors confirm that all experiments were performed under relevant guidelines and regulations. Written Informed consent was obtained from each participant and confidentiality was granted for the information collected from each study participants.

## Data Availability

The datasets analyzed during the current study were available from the corresponding author upon reasonable request.
